# Comprehensive analysis of lysine lactylation in *Frankliniella occidentalis*

**DOI:** 10.3389/fgene.2022.1014225

**Published:** 2022-11-01

**Authors:** Dong An, Liyun Song, Ying Li, Lili Shen, Pu Miao, Yujie Wang, Dongyang Liu, Lianqiang Jiang, Fenglong Wang, Jinguang Yang

**Affiliations:** ^1^ Key Laboratory of Tobacco Pest Monitoring, Controlling and Integrated Management, Tobacco Research Institute of Chinese Academy of Agricultural Sciences, Qingdao, China; ^2^ Luoyang City Company of Henan Province Tobacco Company, Luoyang, China; ^3^ Liangshan State Company of Sichuan Province Tobacco Company, Mile, China

**Keywords:** lysine lactylation, western flower thrips (*Frankliniella occidentalis*), post-translational modification, ribosome, carbon metabolism

## Abstract

Western flower thrips (*Frankliniella occidentalis*) are among the most important pests globally that transmit destructive plant viruses and infest multiple commercial crops. Lysine lactylation (Klac) is a recently discovered novel post-translational modification (PTM). We used liquid chromatography-mass spectrometry to identify the global lactylated proteome of *F. occidentalis*, and further enriched the identified lactylated proteins using Kyoto Encyclopedia of Genes and Genomes (KEGG) and Gene Ontology (GO). In the present study, we identified 1,458 Klac sites in 469 proteins from *F. occidentalis*. Bioinformatics analysis showed that Klac was widely distributed in *F. occidentalis* proteins, and these Klac modified proteins participated in multiple biological processes. GO and KEGG enrichment analysis revealed that Klac proteins were significantly enriched in multiple cellular compartments and metabolic pathways, such as the ribosome and carbon metabolism pathways. Two Klac proteins were found to be involved in the regulation of the TSWV (Tomato spotted wilt virus) transmission in *F. occidentalis*. This study provides a systematic report and a rich dataset of lactylation in *F. occidentalis* proteome for potential studies on the Klac protein of this notorious pest.

## Introduction

Protein post-translational modifications (PTMs) are important cellular regulatory mechanisms. A majority of the proteins are post-translationally modified, and the same proteins can also undergo multiple PTMs, enriching the types and functions of proteins. PTMs endow distinct biological functions to the same proteins, resulting in a diverse functional repertoire of the post-translationally modified protein even if the expression level of the protein does not change (Robertson et al., 2008). The PTM modified proteins participate in various processes, such as protein synthesis and degradation, transcriptional regulation, signal recognition transduction, metabolic regulation, response to biotic and abiotic stress, and other metabolic processes (Goudarzi et al., 2016; Li et al., 2016; Pelletier et al., 2017). Furthermore, PTMs can affect host-pathogen protein interactions by altering the function of the proteins. Network analysis of modified proteins has revealed that PTMs regulate the widely studied virulence factors (Xu et al., 2021). Previous studies have shown that DNA methylation and histone modifications influence the virulence of plant pathogens by altering protein function. Additionally, PTMs also regulate fungal pathogenicity and host response to infections (Dubey and Jeon, 2017; Kong et al., 2018; Nai et al., 2020; Retanal et al., 2021). Plant pathogens attack the immune system by interfering with the phosphorylation state of host proteins, which also enhances pathogen’s pathogenicity (Desclos-Theveniau et al., 2012; Luo et al., 2017). With recent advances in protein separation and mass spectrometry technologies, significant breakthroughs were made in protein modification omics research, and a greater number of PTMs were discovered. A majority of the novel PTMs identified are classified as short-chain lysine acylation modifications (Gao et al., 2020).

Lysine lactylation (Klac) is identified as a novel PTMs. According to previous reports, Klac stimulates the transcription of histone chromatin in human and mouse cells (Zhang et al., 2019a). Besides, Klac influences carbon metabolism and protein synthesis in rice (Meng et al., 2021). Klac also plays an important role in the pathogenicity of *Botrytis cinerea* (Gao et al., 2020). Klac affects energy metabolism and microtubule motor gene expression in *Trypanosoma brucei* (Zhang et al., 2021). Klac plays an important role in *Toxoplasma gondii* invasion of host cells and significantly affects the energy metabolism of *Toxoplasma gondii* (Yin et al., 2022).

Klac is produced by p300 catalyzing the covalent attachment of L-lactic acid-derived lactyl groups to lysine (Zhang et al., 2019a; Cui et al., 2021). The level of Klac correlates with intracellular lactate concentration. L-lactate is one of the crucial intermediate metabolites of glucose metabolism and is used as mobile fuel for aerobic metabolism. L-lactate can mediate redox reactions between different compartments and is involved in cell survival and gene expression as a signaling molecule (Brooks, 2002; Hashimoto et al., 2007; Passarella et al., 2008; Tauffenberger et al., 2019). Klac was observed in the histone and non-histone proteins in mammalian cells and plant-fungal pathogens (Gao et al., 2020). Previous studies have shown that inhibition of glycolysis can reduce Klac levels. Exogenous glucose may also affect cellular lactylation levels (Hagihara et al., 2021). However, the systematic analysis of Klac modifications in insects has not been much explored.

The species of thrips are rich and diverse. According to incomplete statistics, there are more than 7,700 species of thrips in the world, of which about 1% of thrips species cause damage to crops (Healey et al., 2017). There are four main species of these harmful thrips: Western flower thrips (*F. occidentalis* Pergande), onion thrips (*Thrips tabaci* Lindeman), melon thrips (*T. palmi* Karny), and yellow tea thrips (*Scirtothrips dorsalis* Hood) (Reitz et al., 2020). Thrips feed on various food sources, such as melons, fruits, vegetables, and plants, and they hide in different host plant parts.

In addition to direct feeding on tissue sap, resulting in a large-scale reduction in plant yields, thrips can also transmit various viruses to the host plant, resulting in fatal damage. Previous studies have reported that the secondary damage caused by thrips to plants by transmitting viruses is more severe than the damage caused by direct feeding (Whitfield et al., 2005; Park et al., 2010). Out of these viruses, tomato spotted wilt virus (TSWV), which causes serious economic losses, ranks second among the top ten harmful plant viruses globally, second only to tobacco mosaic virus (TMV) (Schoelz et al., 2011). *F. occidentalis* is the most important transmission vector of TSWV.

In Europe, the migration and distribution of *F. occidentalis* led to the prevalence and outbreak of TSWV disease (de Haan et al., 1992; Wijkamp et al., 1993; Chatzivassiliou, 2008). *F. occidentalis* can only be infected with TSWV at the larvae stage but not at the adult stage. Also, *F. occidentalis* larvae cannot transmit TSWV immediately after acquiring the virus (Wijkamp et al., 1993; Pappu et al., 1998). The TSWV must replicate and multiply in the *F. occidentalis* for more than 72 h before transmission. After successfully acquiring TSWV, *F. occidentalis* carry it for the lifetime (Jones, 2005). However, PTMs in *F. occidentalis* proteins, especially the lactylation modification, have not been explored.

In this study, we used Gene Ontology (GO) and the Kyoto Encyclopedia of Genes and Genomes (KEGG) to analyze Klac site motifs and modified protein structures, and to analyze protein functions, pathways, cellular localization, and domain enrichment. Protein-protein interaction (PPI) network analysis was performed to study protein interactions and explore proteins involved in viral transmission in *F. occidentalis*. Our study systematically revealed the distribution of Klac in *F. occidentalis* for the first time and provided important data support for the control of *F. occidentalis*.

## Experimental procedures

### Insect rearing

Western flower thrips (*F. occidentalis*) were collected from the clover plants (*Trifolium repens* L.) in 2018 from the experimental site located at Qingdao Agricultural University (N 36°31′, E 120°39′). These thrips were grown in glassware in a lighted incubator at 28°C, fed regularly with lentil pods, and the incubator light cycle was set to 16 h light and 8 h dark. The thrips mixed samples containing the four developmental stages of egg, larva, pupa, and adult were collected in centrifuge tubes (60 mg per tube, three tubes in total) for protein extraction.

### Protein extraction and trypsin digestion

Thrips samples ground in liquid nitrogen were placed in buffer (1% protease inhibitor (Cat# MBP003, Micron Biotechnology Co., Ltd., Hangzhou, China), 10 mM dithiothreitol, 1% Triton X-100, 8 M urea, and 50 μM PR-619) on ice and sonicated using a sonicator (set the sonicator (Scientz, Ningbo, China) to 30% power, sonicate for 3 s, stop for 5 s, continue for 2 min, and repeat the process three times). After centrifugation at 20,000 g for 10 min at 4°C, the supernatant was collected and pre-cooled 15% trichloroacetic acid (TCA) was added, and the reaction was carried out at −20°C for 2 h. After centrifugation at 20,000 g for 10 min at 4°C, the supernatant was discarded, the pellet was washed three times with acetone and redissolved in buffer (100 mM Ammonium carbonate, 8 M urea, pH 8.0). To digest proteins, the solution was reduced with 10 mM DTT for 1 h at 37°C, followed by alkylation with 20 mM iodoacetamide (IAA) for 45 min at 25°C, protected from light. To eliminate the effect of urea on trypsin digestion, add 100 mM Ammonium carbonate to dilute the urea concentration to 2 M, the first digestion overnight is to mix trypsin to protein mass ratio 1:50, the second digestion takes 4 h and trypsin to protein mass ratio of 1 :100. We used the GE Healthcare 2-D Quant Kit (SKU: 80-6483-56) to determine protein mass.

### Affinity enrichment

To purify Klac peptides, tryptic peptides were incubated with Pan Anti-L-Lactyl Lysine Rabbit pAb (Cat#WM101, Micron Biotechnology Co., Ltd., Hangzhou, China) in NETN buffer (0.5% NP-40, 50 mM Tris-HCl, 1 mM EDTA, 100 mM NaCl, pH 8.0) overnight at 4°C. The beads were washed four times with NETN buffer and twice with ddH_2_O. The Klac peptide on the beads was eluted with 0.1% TFA and dried under vacuum. Final analysis using liquid chromatography-tandem mass spectrometry (LC-MS/MS).

### LC-MS/MS analysis

The peptides were solubilized with 300 μl of 0.1% formic acid, loaded onto a reversed-phase pre-column, and separated using a reversed-phase analytical column. First complete linear gradient over 24 min with 6%–22% solvent B (0.1% FA in 98% acetonitrile (ACN)), then 8 min with 22%–40% solvent B, ramp up to 80% over 5 min and hold at 80% over the last 3 min, and maintained a constant flow rate of 300 nl/min on the EASY-NLC1000UPLC system. The washed peptides were analyzed by a Q Exactive^TM^ Plus Hybrid Quadrupole-Bitrap mass spectrometer. Peptides were placed under a nanospray (NSI) source and connected online to UPLC for tandem mass spectrometry (MS/MS) using a Q Exactive™ Plus MS. The Orbitrap was adjusted to a resolution of 70,000 for detection of intact peptides. Peptides were selected for MS/MS with Normalized Collision Energy (NCE) set to 30. The ion fragments were detected in orbit with a resolution of 17,500. For the first 20 progenitors with ion counts above the 5 × 10^3^ threshold in the MS overview scan, a data correlation method was used to switch between 1 MS scan and 20 MS/MS scans with a dynamic cutoff of 15.0 s. The electrospray voltage was set to 2.0 kV. Automatic gain control (AGC) was used to avoid overfilling the Orbitrap until 5 × 10^4^ ions were accumulated to generate the correct MS/MS spectrum. The MS scan range was set from 350 to 1800 m/z and the initial mass was fixed at 100 m/z.

### Data analysis

MS/MS data were retrieved using the MaxQuant search engine (v.1.4.2). Erroneous tandem mass spectra were excluded by adding a reverse contamination database. The cleavage enzyme was trypsin/P and the maximum missing value was set to 4, the maximum number of labeled amino acids was set to 5, the minimum length was set to 7 amino acid residues, and the maximum number of peptide modifications was set to 5. Mass errors were set to 10 ppm (error of parent ions) and 0.02 Da (error of fragment ions). Aminomethylation of Cys was a fixed modification, oxidation of Met was a variable modification, and lactylation of Lys and lactylation of the N-terminus of proteins are variable modifications. A 1% false detection rate (FDR) threshold was set for proteins, peptides, and modification sites.

### Bioinformatics analysis

All database protein sequences were set as background parameters, and the rest of the parameters were kept unchanged by default, and the amino acid sequence model around 21-mers (10 amino acids upstream of the site and 10 amino acids downstream of the site) in the modified protein sequence was generated by MoMo program (V5.0.2) (Chou and Schwartz, 2011). The MOMO algorithm was set to motif-x, the minimum number of occurrences was set to 20, and the *p*-value threshold was 0.000001. Protein secondary structure analysis was performed using NetSurfP (Klausen et al., 2019). The subcellular localization of lactylalted proteins was predicted, classified and analyzed by WOLF PSORT software.

Gene Ontology (GO) annotation was used to categorize proteins into three broad categories, i.e., biological processes, intracellular compartments, and molecular functions. For each category, a two-tailed Fisher’s exact test was used to test the significance of the identified protein enrichment for all database proteins. Fixed multiple assumptions for testing false detection rates with standard controls were also used. The GO with a corrected *p*-value < 0.05 was considered to be significant. The identified proteins were GO-annotated using the UniProt-GOA database (http://www.ebi.ac.uk/GOA/). These protein IDs were first converted to UniProt IDs, which in turn were mapped to GO IDs. If the identified protein has no annotation information in the UniProt-GOA database, the GO function of the protein was predicted using InterProScan v.5.14-53.0 (http://www.ebi.ac.uk/interpro/).

Kyoto Encyclopedia of Genes and Genomes (KEGG) database was used to determine the significance of enriched proteins *versus* all proteins in the database using a two-tailed Fisher’s exact test. Fixed multiple assumptions were used for testing false detection rates by implementing standard controls. Pathways with post-test *p*-value < 0.05 were judged to be significantly different. These pathways were categorized using the KEGG website. The InterPro database which can be used to classify protein families, predict protein domains and modification sites, and analyze protein sequence function was used to study each protein class obtained, as well as a two-tailed Fisher’s exact test—using a long list of rigorous probabilistic tests of the key methods for identifying proteins were applied to all databases. Correction for multiple hypothesis testing was carried out using standard FDR control methods, and domains with a corrected *p*-value < 0.05 were considered to be significant. The STRING database of Protein-Protein Interaction Name Identifier Version 10.0 (http://string-db.org/) was searched for all proteins with Klac modifications (associated with the ribosome pathway). External candidates were excluded because only interactions between proteins belonging to the study dataset were selected. STRING defined metric “trust score” was used to determine the trust in an interaction. Cytoscape (version 3.3.0) aggregated all interactions with trust scores ≥0.9 in the STRING interaction network. MCODE (Molecular Complex Detection) was used to analyze densely connected regions of graphs for theoretical clustering algorithms.

## Results

### Lysine lactylation proteomic analysis of *Frankliniella occidentalis*

**FIGURE 1:**
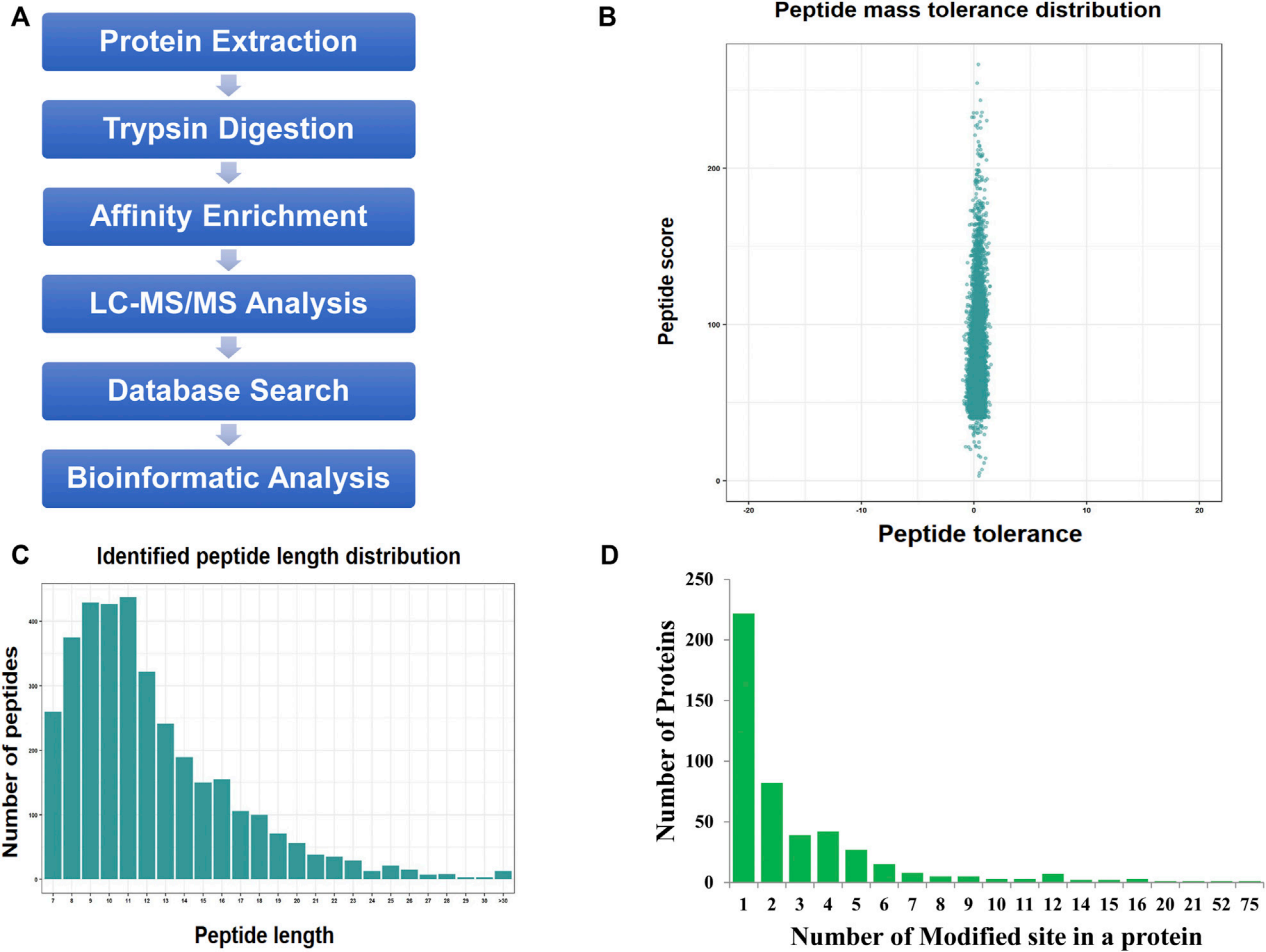
Protein lysine lactylation assay of *F. occidentalis*. **(A)** Technical route of Klac assay. **(B)** Mass error range for all identified Klac peptides (The error distribution for Klac peptides is close to 0, and the vast majority are less than 0.02 Da). **(C)** Klac peptide length range (Most of the peptides are distributed in 7–20 amino acids, which conform to the general rules based on trypsin enzymatic hydrolysis and HCD fragmentation). **(D)** Number of Klac sites in the protein of *F. occidentalis* (Most of the Klac proteins have less than three modification sites).

We used a proteomic approach involving affinity enrichment and mass spectrometry to identify the complete lactylome of *F. occidentalis* and map the associated Klac sites (Figure 1A). A total of 1,458 Klac sites were identified in 469 Klac proteins (Supplementary Tables S1,S2). A total of 20,885 proteins were estimated to be encoded by the *F. occidentalis* genome, indicating that proteins with Klac modification accounted for 2.2% (469/20,885) of the *F. occidentalis* proteome. Validation of mass errors for all detected Klac peptides revealed that the error distribution was close to 0, and the vast majority were <0.02 Da, implying that our MS data were reliable (Figure 1B). The properties of the screened Klac peptides were consistent with those of the trypsin peptides and met the standard, and their lengths were mostly between 8 and 20 (Figure 1C). For all detected Klac proteins, 47.3% (222/469) contained one modification site, 17.5% (82/469) and 8.3% (39/469) contained two or three modification sites, in addition, we also found that 4.5% (21/469) of the Klac proteins had more than ten modification sites (Figure 1D; Supplementary Table S3).

### Structural analysis of lysine lactylation proteins and motif analysis of lysine lactylation sites in *Frankliniella occidentalis*

**FIGURE 2:**
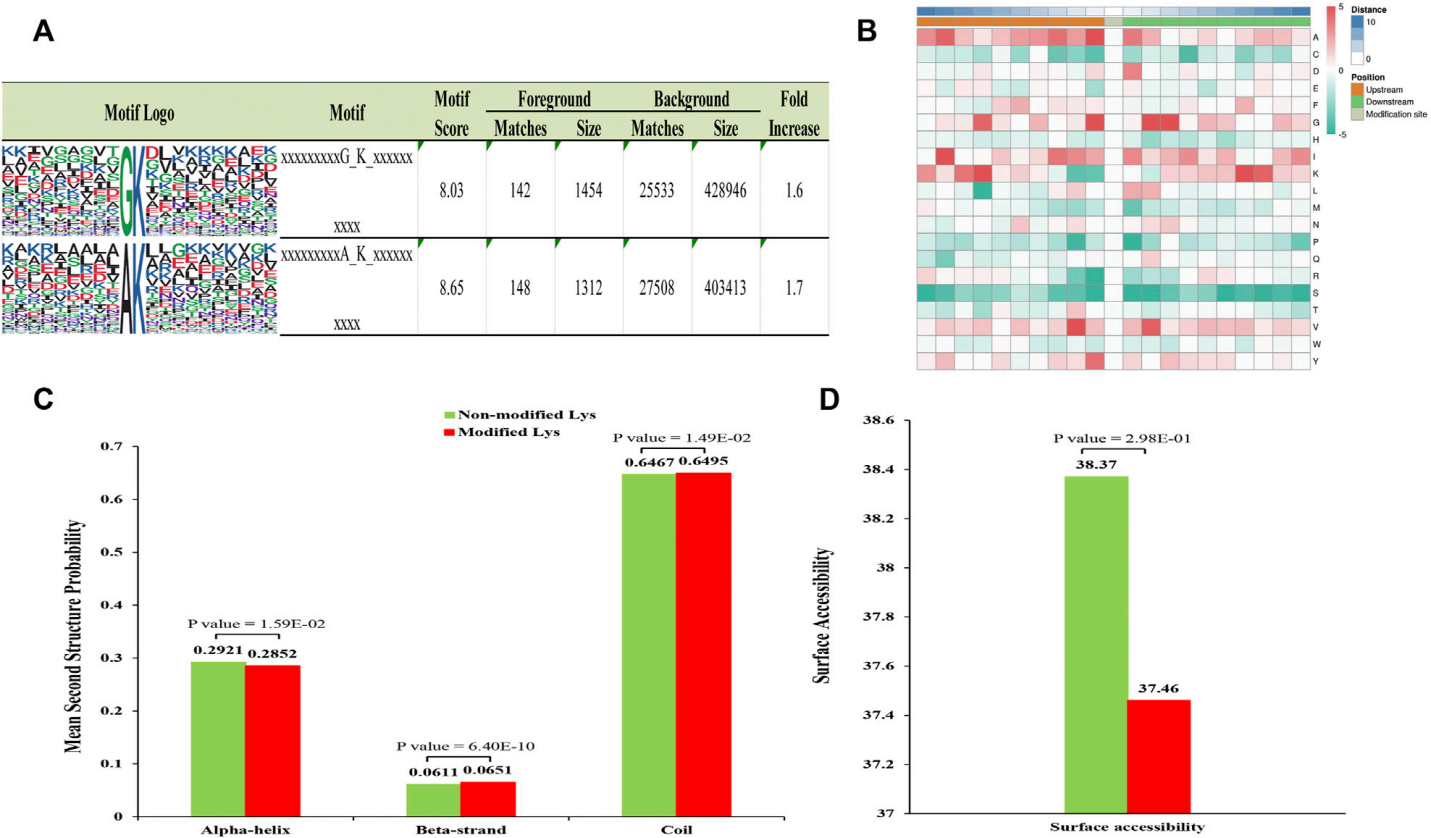
Analysis of related properties of Klac peptides in *F. occidentalis*. **(A)** Analysis of specific amino acid enrichment ± 10 flanking the Klac site (position 0). Lactylation motifs were constructed using the MOMO program. The central K (position 0) represents lactated lysine. Letters of different heights represent the frequency of occurrence of a particular amino acid. **(B)** The heat map represents the degree of enrichment of amino acids around the Klac site. Red indicates high frequency and green means low frequency. **(C)** Identification of the frequency of occurrence of different secondary structures (α-helix, β-strand and coil structures) in the resulting Klac proteins. **(D)** Surface accessibility of predicted Klac sites.

MoMo program was used to evaluate specific amino acid sequence motifs around the Klac sites. A total of four conserved motifs in the lactylome around Klac sites were detected (Figure 2A; Supplementary Table S4). These motifs were identified in the ten amino acids upstream and downstream of Klac sites (−10 Klac +10) in the lactylalted peptides, accounting for 83.2% (3,081/3,503) of all identified peptides. Amino acid frequencies flanking the Klac site were enriched and analyzed as follows (Figure 2B): alanine (A) at −1 position; glycine (G) at −1, +2 and +3 positions; and +3; −7, +7 positions of lysine (K). These sites were found to be favorable for lactylation. The GKlac motifs were found in *Botrytis cinerea* (Gao et al., 2020) and the AKlac motifs also were found in *Toxoplasma gondii* (Yin et al., 2022).

Furthermore, to determine the preferred secondary structure of Klac sites in proteins, we used NetSurfP for processing analysis. It was found that 64.7% of Klac sites were in disordered helices, 29.2% in α-helices, and the rest in β-sheets. The distribution of the lactylalted lysine was not significantly different from that of non-modified lysine (Figure 2C). However, the surface accessibility of non-modified sites was higher than that of Klac sites (Figure 2D), suggesting selective modification of proteins for different biological processes.

### Functional annotation and cellular localization of lysine lactylation proteins in *Frankliniella occidentalis*

**FIGURE 3:**
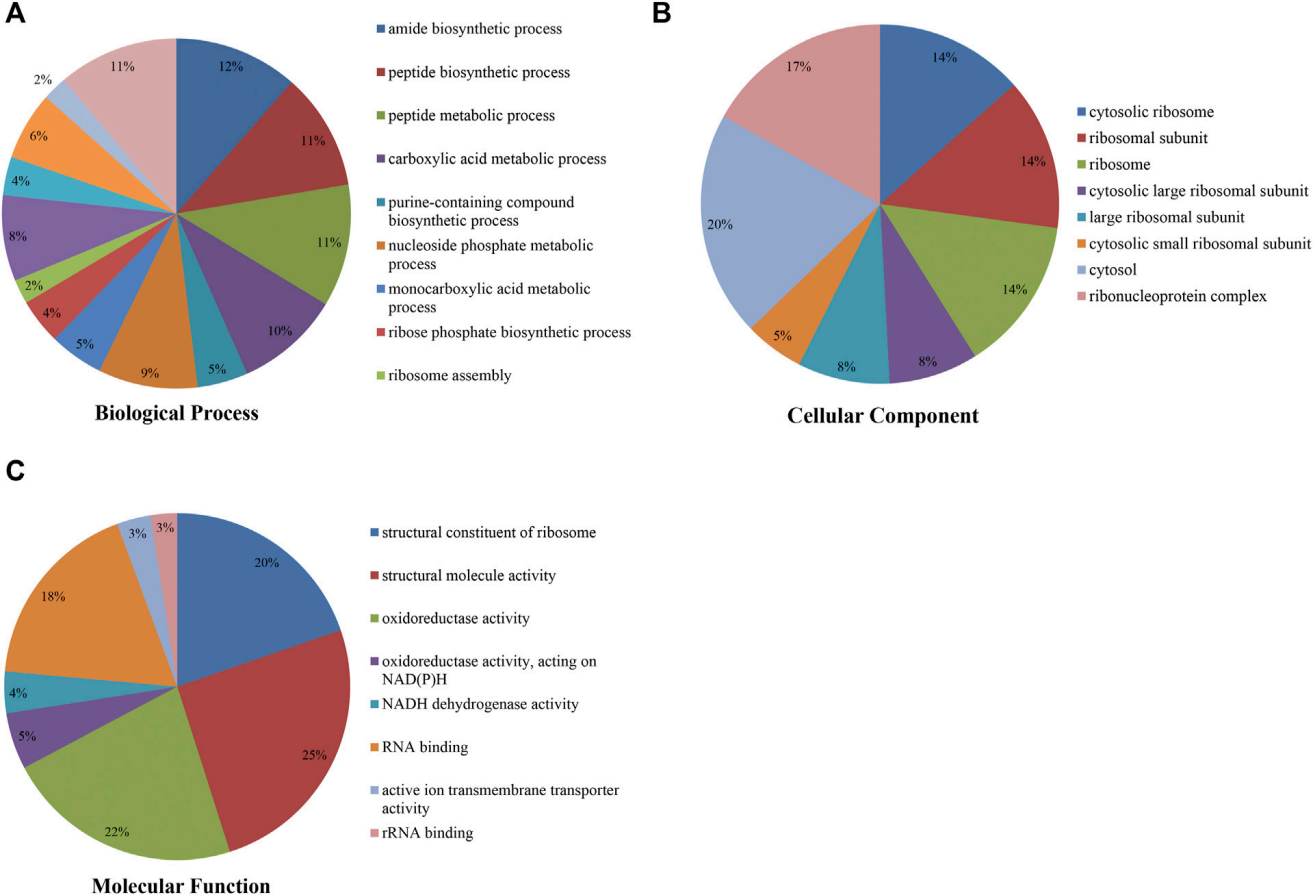
Pie chart showing Gene Ontology analysis of *F. occidentalis* proteins with Klac modifications. **(A)** Klac proteins classified according to biological process. **(B)** Klac proteins classified according to cellular component. **(C)** Klac proteins classified according to molecular function.

To study the Klac modification in *F. occidentalis* proteins, the proteins with Klac modification were subjected to the GO functional analysis (biological process, cellular component, and molecular function) (Figures 3A–C; Supplementary Table S5). In the GO biological process analysis, most of these proteins with Klac modification were enriched in amide biosynthetic (12%) (Figure 3A) and peptide biosynthetic (11%) processes. In the GO cellular component analysis, 20% and 17% of the proteins with Klac modification were enriched in the cytosol and ribonucleoprotein complex, respectively (Figure 3B). In the GO molecular function analysis of proteins with Klac modification, a majority of these proteins were enriched in structural molecule activity (25%), oxidoreductase activity (22%), structural constituent of ribosome (20%), and RNA binding (18%) (Figure 3C). In the subcellular localization analysis, most of the proteins with Klac modification were enriched in the cytoplasm (40.0%), nucleus (19.4%), cytoskeleton (13.5%), endoplasmic reticulum (12.8%), extracellular (5.1%), mitochondria (3.2%), nucleus (3.0%), and plasma membrane (0.9%) (Supplementary Table S6).

### Kyoto encyclopedia of genes and genomes pathway and domain enrichment analyses of lysine lactylation proteins in *Frankliniella occidentalis*

**FIGURE 4:**
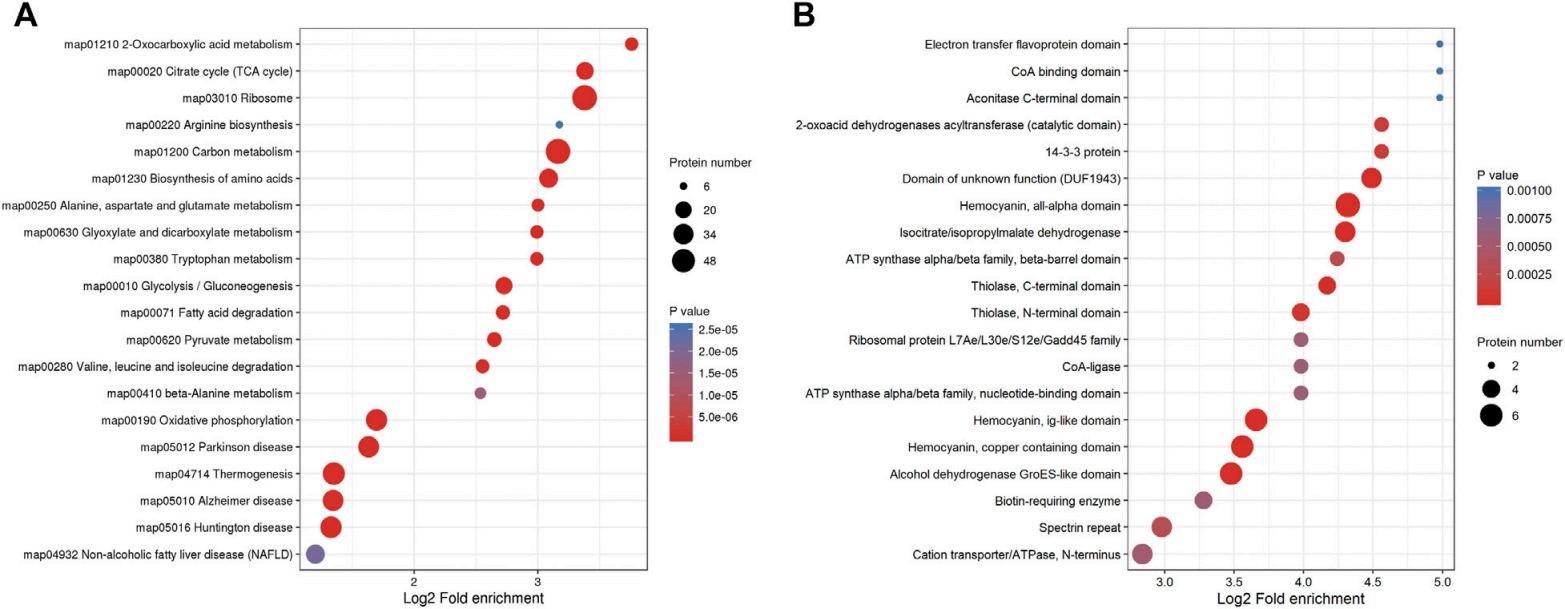
Systematic enrichment analysis of Klac proteins in *F. occidentalis*. **(A)** Klac proteins enrichment analysis based on the KEGG pathway. **(B)** Protein domain-based Klac proteins enrichment analysis.

In order to explore the types and abundance of Klac proteins, we systematically analyzed *F. occidentalis* Klac proteins using KEGG pathway and protein domain enrichment. In KEGG pathway enrichment analysis, the majority of the proteins were enriched in the ribosomes, carbon metabolism, biosynthesis of amino acids, the citrate cycle (TCA cycle), glycolysis/gluconeogenesis, 2-oxocarboxylic acid metabolism, oxidative phosphorylation, Parkinson disease, thermogenesis, the pyruvate metabolism, fatty acid degradation, tryptophan metabolism, glyoxylate, dicarboxylate metabolism, and so on (Figure 4A; Supplementary Table S7). At the same time, the results of protein domain enrichment were consistent with the results of KEGG pathway analysis, we found that many protein domains involved in energy metabolism and stress defense response were lactylalted modified, such as the isocitrate/isopropylmalate dehydrogenase and alcohol dehydrogenase GroES-like domain, hemocyanin, and all-alpha domain (Figure 4B; Supplementary Table S8). These results showed that Klac modifications may affect the role of *F. occidentalis* proteins in metabolic pathways, biosynthesis and adaptation to stress.

### Lysine lactylation proteins in *Frankliniella occidentalis* are involved in central metabolism

**FIGURE 5:**
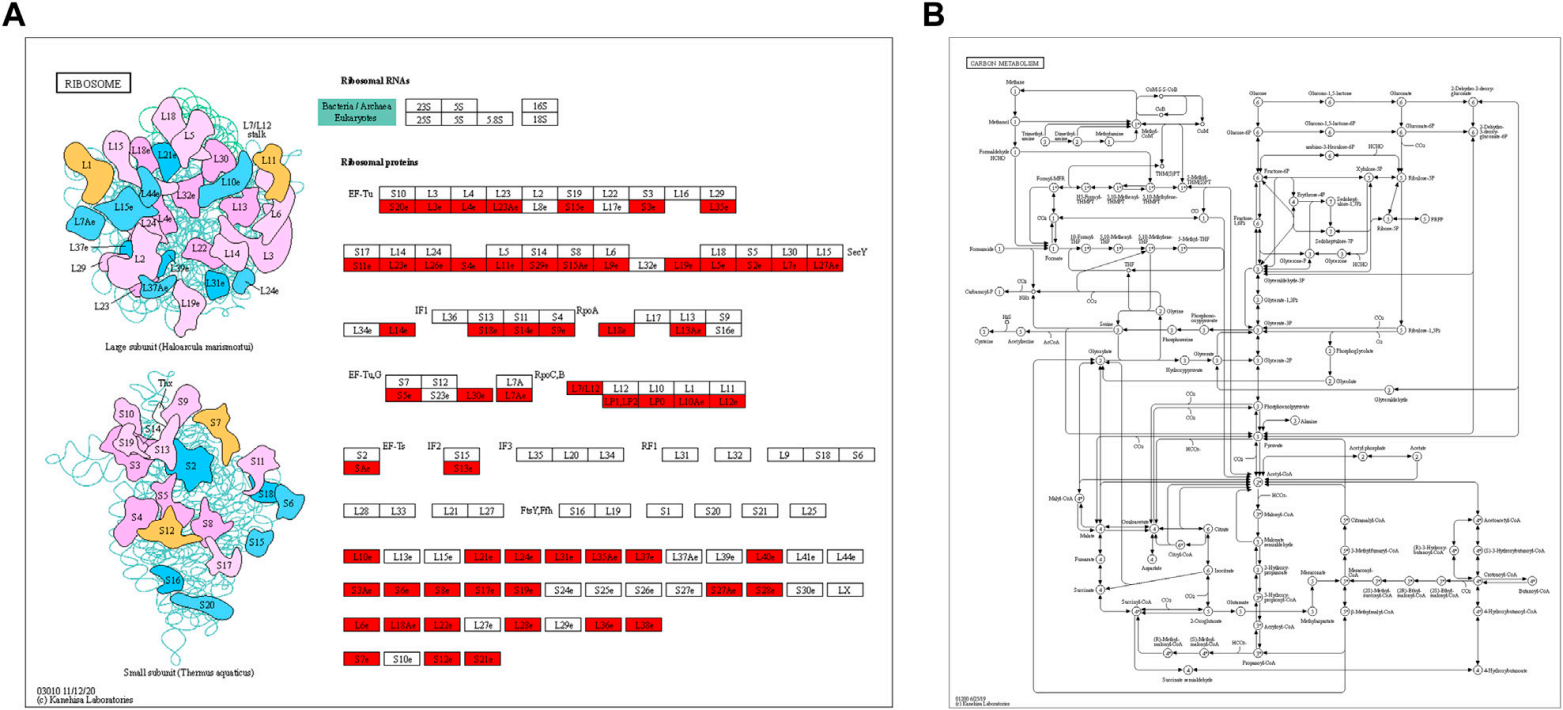
KEGG pathway enrichment analysis of proteins with Klac modifications. **(A)** Klac proteins were involved in the ribosomes. **(B)** Klac proteins were involved in carbon metabolism. Proteins with Klac modifications are highlighted in red.

Through KEGG pathway enrichment analysis, we found that many proteins related to ribosomes and carbon metabolism were Klac modified. In ribosomes, half of the large subunit and small unit proteins were found to have Klac modifications at several sites (Figure 5A). Carbon metabolism is an important metabolic process that provides the necessary carbon framework and energy to synthesize amino acids, proteins, and nucleic acids. We observed that lactylation was widespread in various proteins involved in the carbon metabolism (Figure 5B). Therefore, this means that Klac modification affects the energy metabolism and protein synthesis of *F. occidentalis*.

### Protein interaction network analysis

**FIGURE 6:**
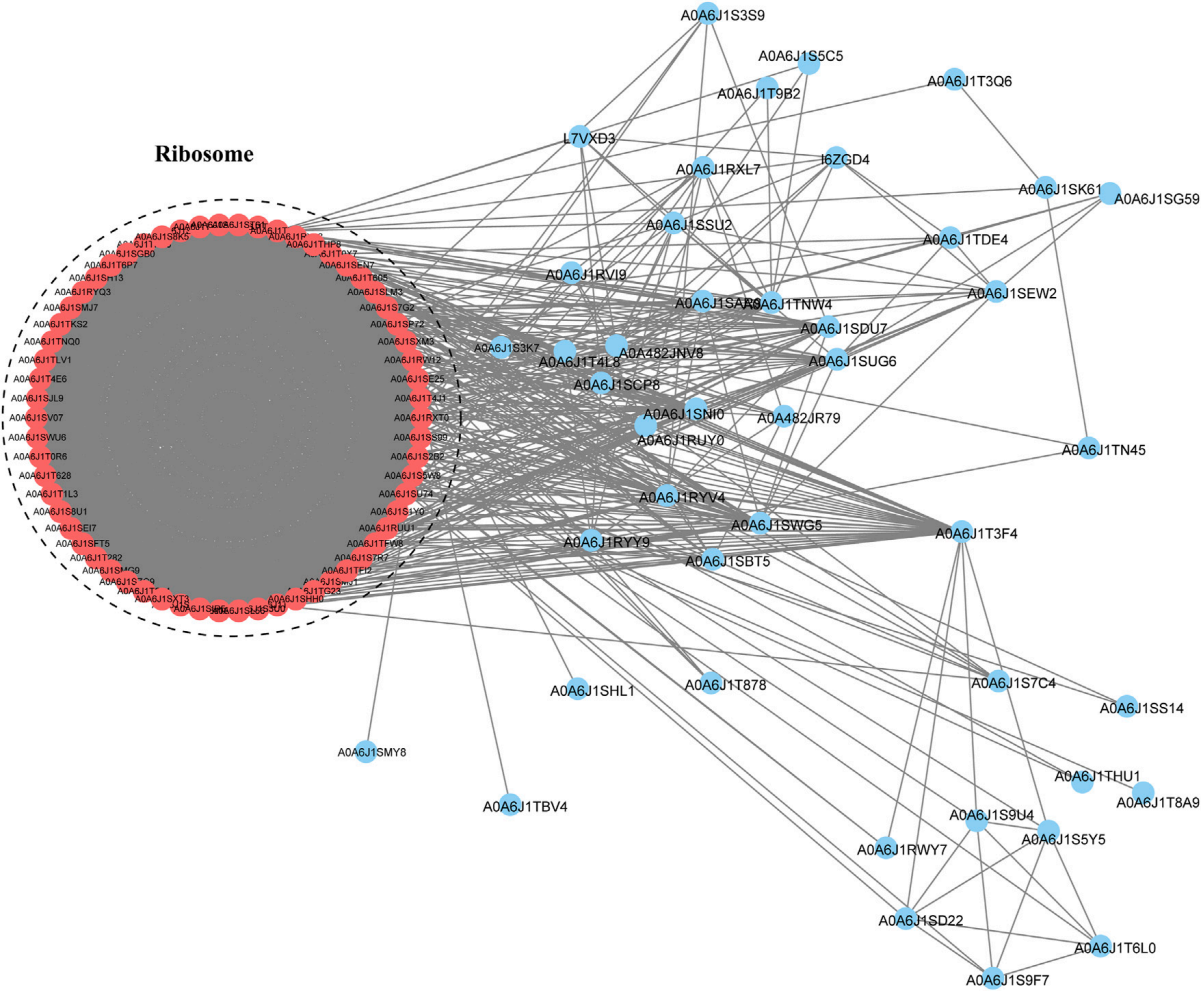
Protein-protein interaction (PPI) network analysis of Klac proteins in *F. occidentalis*. The interaction network of STRING was visualized in Cytoscape (version 3.3.0). The size of the nodes corresponds directly to the number of Klac sites per protein. This group of proteins was highly associated with ribosomes.

To study the interactions between proteins in multiple molecular processes, we constructed a PPI network of Klac proteins in *F. occidentalis* using the STRING database (Figure 6; Supplementary Table S9). A total of 106 Klac proteins were enriched for this network and these proteins were found to mainly affect the ribosomal pathway. This result was consistent with GO and KEGG pathway enrichment, suggesting that the enriched Klac proteins were involved in specific pathways in *F. occidentalis*.

### Analysis of lysine lactylation proteins involved in the mechanism of tomato spotted wilt virus transmission by *Frankliniella occidentalis*

**FIGURE 7:**
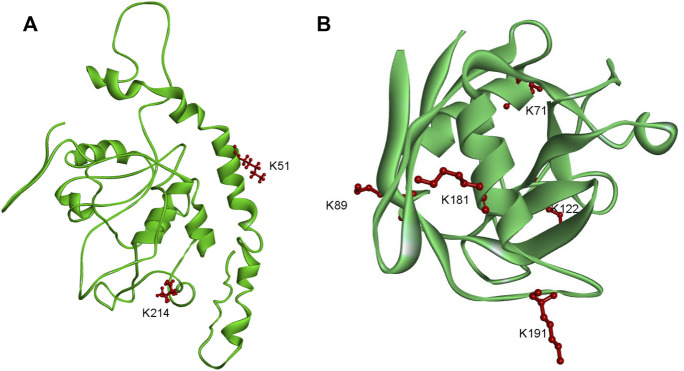
Schematic representation of the three-dimensional structures of two proteins in *F. occidentalis* involved in the transmission of TSWV. **(A)** EndoCP-GN. **(B)** Cyclophilin. Structure predicted by I-TASSER. Identified Klac sites were highlighted in red.

*F. occidentalis* is an important virus transmission vector. The data showed that the two proteins involved in the mechanism of TSWV transmission by *F. occidentalis* were lactylated. EndoCP-GN (GenBank accession: MH884757) showed two modification sites (K51 and K214) (Figure 7A), and the location of this protein was the midgut and salivary glands of *F. occidentalis*. Besides, it interacts with the glycoprotein of TSWV and affects TSWV infection of *F. occidentalis*. (Badillo-Vargas et al., 2019). We also found another protein cyclophilin (GenBank accession: MH884760), with five modification sites (K71, K89, K122, K181, and K191) (Figure 7B). This protein is involved in metabolic activities and is closely related to TSWV-GN interaction (Badillo-Vargas et al., 2019). Cyclophilin has been shown to prevent viral infections and play a vital role in the transmission of grain yellow dwarf virus *via* aphids as a carrier (Liu et al., 2018; von Hahn and Ciesek, 2015; Tamborindeguy et al., 2013). These findings suggested that Klac protein might be involved in viral transmission and energy metabolism in *F. occidentalis*, in line with the GO and KEGG enrichment analysis data.

## Disscussion

Klac is the newly identified PTM, which is involved in multiple biological functions. Klac has been found in a few species, engineered humans, mice, rice, and *Botrytis cinerea* (Zhang et al., 2019a; Gao et al., 2020; Meng et al., 2021). Till now, protein lactylation in insects, especially *F. occidentalis* has not been explored. *F. occidentalis* is an important pest of cultivated crops. Thus, in this study, for the first time, we have systematically investigated the lactylation of proteins in *F. occidentalis*.

We identified a total of 1,458 lactylation sites in 469 proteins using liquid chromatography-tandem mass spectrometry (LC-MS/MS). *F. occidentalis* proteins with Klac modification accounted for 2.2% of the total proteome of *F. occidentalis*. Analysis of the secondary structure of *F. occidentalis* proteins showed that Klac sites were located in the secondary structures of different proteins, which indicated that Klac modification was widely distributed in all proteins. We also observed that Klac modifications primarily occur in α-helical structures than in β-sheet structures. The α-helical structure is the most abundant secondary structure in proteins and is involved in various functions, including DNA binding, protein interactions, and membrane protein stability (McKay et al., 2018; Manav et al., 2019, P et al., 2019). Therefore, this result suggests that Klac may be involved in the above-mentioned biological functions.

The Subcellular localization results indicated that Klac proteins were distributed in multiple intracellular compartments, belonging to multiple functional groups, and may play unique and important roles in regulating various cellular processes in *F. occidentalis*. In addition, a myriad of Klac proteins is involved in cellular and metabolic processes, suggesting that Klac may be involved in the cellular development and metabolic activities in *F. occidentalis*. Besides, proteins with Klac modifications are also associated with ribosomes, a vital organelle for protein synthesis. Previous studies have shown that PTMs are crucial for the biological function of ribosomal proteins. For instance, N-acetylation of ribosomal proteins significantly affects the protein synthesis of yeast ribosomes (Kamita et al., 2011). Differential translation of human-specific mRNAs is regulated *via* phosphorylation of the ribosomal protein Rps15/Us19 (D and M, 2017). Thus, the outcomes of this study suggest that lactylation of ribosomal proteins may play an important role in their functions. In addition, proteins with Klac modification were also found to be involved in carbon metabolism, amino acid biosynthesis, glycolysis, TCA, oxidative phosphorylation, pyruvate metabolism, tryptophan metabolism, glyoxylate metabolism, fatty acid degradation, and so on. This suggests that lactylation significantly affects the metabolic pathwaysand biosynthesis aspects of *F. occidentalis*.

Protein domain enrichment analysis revealed that many proteins affecting stress defense responses and energy metabolism were lactylalted, including hemocyanin. Hemocyanin transports oxygen in the hemolymph of various arthropods and affects the interaction of crucifers with the diamondback moth (*Plutella xylostella*) (Burmester, 2002; Zhang et al., 2019b). Hemocyanin could attenuate the infection of penaeid shrimp by WSSV (White spot syndrome virus) and enhance the antibacterial immune response of Penaeid shrimp (Zhan et al., 2019; Aweya et al., 2022). Hemocyanin also affects the metamorphosis, reproduction, and development of Beet armyworm (*Spodoptera exigua*) (Tang et al., 2010). This study suggests that Klac proteins might be involved in developmental processes, affecting energy metabolism, growth and development, and the ability to cope with biotic and abiotic stresses in thrips.

To identify the conservation of Klac sites across species, we first compared Klac sites of *F. occidentalis* with Klac sites of *Oryza sativa* and *Botrytis cinerea*. This resulted in the identification of conserved lysine sites in Klac. We identified a total of 1,314 Klac sites in *F. occidentalis* in the two species. The number of conserved Klac sites in *Oryza sativa* and *Botrytis cinerea* were 340 and 355, accounting for 54% and 51% respectively (Supplementary Table S10). We also observed 117 conserved Klac sites in two organisms (Supplementary Table S10). This data suggests that in addition to many conserved Klac sites that may regulate proteins involved in similar regulatory pathways and cellular processes, different species may have unique Klac sites, imparting specific physiological functions to Klac proteins.

Intermediate vector-borne diseases caused by viruses that infect plants and animals are one of the most important agricultural problems worldwide (Gubler, 2002). Most viruses infecting plants are transmitted by arthropods. As the most important transmission vector of TSWV, *F. occidentalis* causes devastating losses to economic crops worldwide. TSWV synthesizes a structural glycoprotein (GN) for viral attachment and viral expression in arthropod vector host cells. A previous study reported that two proteins, cyclophilin and endocuticle structural glycoprotein (EndoCP-GN), interact with the GN of *F. occidentalis* (Badillo-Vargas et al., 2019). We found that these two proteins have multiple Klac sites, suggesting that lactylation may play an important role in mediating the transmission of TSWV by *F. occidentalis*. Therefore, exploring the link between the Klac proteins of *F. occidentalis* and TSWV may lead to the development of new strategies for the control of TSWV transmission. Furthermore, cuticular proteins and cyclophilins have been shown to be involved in various viral infection processes.

Previous studie has shown that the CP of the hemipteran vector *Laodelphax striatellus* interacts with the nucleocapsid protein (pc3) of rice streak virus (genus *Tenuivirus*, family *Phenuiviridae*), which affects virus transmission (Liu et al., 2015). A recent study has reported interaction between the CP of another hemipteran vector, *Rhopalosiphum padi,* with the readthrough protein of the barley yellow dwarf virus-GPV (genus *Luteovirus*, family *Luteoviridae*), which influences the aphid transmission of the barley yellow dwarf virus-GPV (Wang et al., 2015). In addition, it has been reported that cyclophilin A can affect the replication of tomato bushy stunt virus (genus *Tombusvirus*, family *Tombusviridae*) (Kovalev and Nagy, 2013) by interacting with viral RNA, and the aphid vector *Schizaphis graminum* cyclophilins have been reported to be efficient transmitters of cereal yellow dwarf virus (genus *Polerovirus*, family *Luteoviridae*) (Tamborindeguy et al., 2013). The current study has shown that the cyclophilin and endocuticle structural glycoprotein (EndoCP-GN) undergo lactylation, suggesting that lactylation may be involved in this process. These findings have important implications in developing plant pest management and protection against intermediate host-transmitted viruses.

## Conclusion

In conclusion, this study presents a global map of lactylation in *F. occidentalis*. This study reveals that proteins with Klac modification play crucial roles in biosynthesis, energy metabolism, and adaptation to stress. Besides, this study also provides two candidate proteins that might be involved in the virus transmission and control strategy of *F. occidentalis*. Thus, studying the lactylation function of the target protein might help with the prevention and control *F. occidentalis*.

## Data Availability

The data presented in the study are deposited in the ProteomeXchange Consortium repository (https://www.ebi.ac.uk/pride/archive), accession number PXD030799.
